# Gout tophi on the soles resembling viral warts

**DOI:** 10.1002/ccr3.1539

**Published:** 2018-04-24

**Authors:** Simon Bossart, Daniel Sidler, Roland Blum, Robert E. Hunger

**Affiliations:** ^1^ Department of Dermatology, Inselspital Bern University Hospital University of Bern Bern Switzerland; ^2^ Department of Nephrology, Hypertension, Inselspital Bern University Hospital University of Bern Bern Switzerland

**Keywords:** Common warts, cutaneous tophi, gout, gout tophi, tophaceous gout, viral warts

## Abstract

This case highlights the need to consider tophaceous gout in patients with post‐transplant renal insufficiency, chronic immunosuppression, and hypertension, who develop atypical papules or nodules on the soles, which can cause problems in differentiation from other skin diseases.

## Case Report

Gout is a systemic disease that evolves from the deposition of monosodium urate crystals in tissues. These crystals can be accumulated and deposited in all tissues, forming tophi 1. Tophi typically occur around the joints, in the subcutaneous or dermal tissue. Frequent locations are the helix of the ear, olecranon bursa, hands, knees, feet, and fingers 2.

As gout tends to present as articular disease first, cutaneous gout forming tophi usually correlate with chronicity and uncontrolled disease. Sometimes, skin involvement appears as small, superficial, whitish and sometimes pustule‐like lesions with increasing pain, swelling, erythema, and a tendency for ulceration 3. There are only few reports of cutaneous tophi of the soles.

We report a 33‐year‐old kidney transplant male patient under long‐term immunosuppression and with a history of hyperuricemia and gout, who was referred to our dermatological outpatient unit with the presentation of whitish skin lesions on the soles of his feet, some of them similar to verrucous warts. The patient's medical history included a kidney transplantation at the age of 12 because of severe reflux nephropathy. He described the development of small painful indurated nodules on both soles within months, preceded by several episodes of inflammatory arthritis in the right knee, right ankle, left shoulder as well as the metacarpophalangeal joints of both hands. Medication included amlodipine, candesartan, tacrolimus, mycophenolic acid, and natriumhydrogencarbonate.

**Figure 1:**
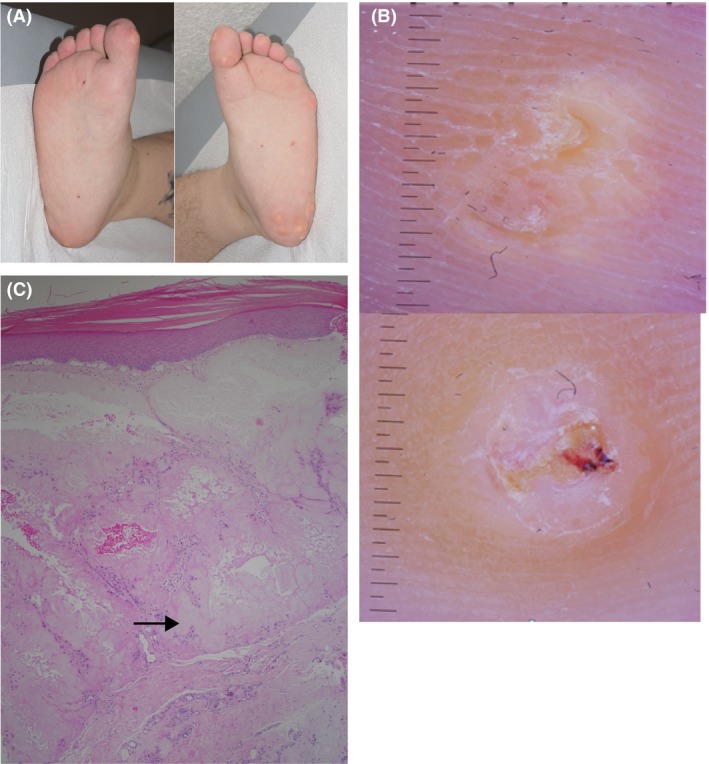
(A) hyperkeratotic nodules and papules on the soles of the feet and toes. (B) Dermoscopy revealed yellowish homogenous annular plaques with central scaly and partially hemorrhagic papules. (C) dermal nodular deposits of amorphous pink material surrounded by a sparse granulomatous inflammation (hematoxylin and eosin stain, ×40 original magnification).

Physical examination revealed hyperkeratotic annular nodules and papules on both soles of the feet and toes, painful on palpation (Fig. 1A). Dermoscopy showed diffuse yellowish homogenous annular plaques with central scaly and partially hemorrhagic papules (Fig. 1B), reminding of verrucous warts. Serum creatinine level was at baseline at 175 *μ*mol/L (range 59–104 *μ*mol/L), estimated glomerular filtration rate was 43 mL/min/1.73 m^2^ (range >59 mL/min/1.73 m^2^), blood urea level was 15.5 mmol/L (range 3.2–7.3 mmol/L), serum urate concentration was 447 *μ*mol/L (range 202–416 *μ*mol/L), and calcium and serum phosphate levels were normal.

Because of its remarkable appearance, a biopsy was taken. Histology revealed dermal nodular deposits of amorphous pink material surrounded by a sparse granulomatous inflammation, consistent with the diagnosis of gout (Fig. 1C, arrow).

Treatment for suspected gout was started before referral and included colchicine 1 mg daily as well as high‐dose glucocorticoids, leading to a rapid clinical improvement within few days.

Chronic gout with tophaceous changes on palms and soles can cause problems in differentiation from other skin diseases 3, 4. Involvement of the hands has been reported in few cases, usually being associated with renal dysfunction and thiazide diuretics 2. To the best of our knowledge, there is only one other report that described a patient who developed abrupt pustular gouty tophi on soles and hands after an episode of arthritis 5.

The present case illustrates the development of tophaceous gout on the soles in a patient with post‐transplant renal insufficiency, chronic immunosuppression, and hypertension. It highlights the need to consider tophaceous gout in patients with atypical papules or nodules on the soles, which sometimes can show deceptive similarities to viral warts. Timely recognition of these features facilitates prompt initiation of urate‐lowering therapy, limiting future gout attacks.

## Conflict of Interest

None declared.

## Authorship

SB: reviewed the patient, performed the literature review and wrote the manuscript. DS, RB: reviewed the patient, reviewed and edited the manuscript. RH: supervised the research project, reviewed the patient, and reviewed and edited the manuscript.
